# Meretoja's Syndrome: Lattice Corneal Dystrophy, Gelsolin Type

**DOI:** 10.1155/2017/2843417

**Published:** 2017-01-31

**Authors:** I. Casal, S. Monteiro, C. Abreu, M. Neves, L. Oliveira, M. Beirão

**Affiliations:** ^1^Centro Hospitalar do Porto, Hospital de Santo António, Porto, Portugal; ^2^Instituto de Ciências Biomédicas Abel Salazar, Universidade do Porto, Porto, Portugal

## Abstract

Lattice corneal dystrophy gelsolin type was first described in 1969 by Jouko Meretoja, a Finnish ophthalmologist. It is caused by an autosomal dominant mutation in gelsolin gene resulting in unstable protein fragments and amyloid deposition in various organs. The age of onset is usually after the third decade of life and typical diagnostic triad includes progressive bilateral facial paralysis, loose skin, and lattice corneal dystrophy. We report a case of a 53-year-old female patient referred to our Department of Ophthalmology by severe dry eye and incomplete eyelid closure. She had severe bilateral facial paresis, significant orbicularis, and perioral sagging as well as hypoesthesia of extremities and was diagnosed with Meretoja's syndrome at the age of 50, confirmed by the presence of gelsolin mutation. At our observation she had bilateral diminished tear film break-up time and Schirmer test, diffuse keratitis, corneal opacification, and neovascularization in the left eye. She was treated with preservative-free lubricants and topical cyclosporine, associated with nocturnal complete occlusion of both eyes, and underwent placement of lacrimal punctal plugs. Ocular symptoms are the first to appear and our role as ophthalmologists is essential for the diagnosis, treatment, and monitoring of ocular alterations in these patients.

## 1. Introduction

Amyloidosis is a group of diseases that can be divided into localized or systemic and the etiology is primary, secondary, or hereditary. Lattice corneal dystrophy, gelsolin type—Meretoja's syndrome—is also called lattice corneal dystrophy type 2 (LCD2) or familial amyloidotic polyneuropathy (FAP) type IV and is an autosomal dominant inherited disease caused by a mutation G654A or G654T in the gelsolin gene at 9q32–34 [1–3]. The biochemical analyses have indicated that the amyloid fibrils formation is related to mutant gelsolin [4] and amyloid deposition is found in various tissues such as cornea, skin, vascular walls, and perineurium [5, 6]. It was first described in 1969 by Dr. Meretoja, a Finnish ophthalmologist [7], and since then some cases in a limited number of countries have been reported [8], probably because the disease is underdiagnosed or misdiagnosed [9–11]. The age of onset is usually after the third decade of life and the first sign of the disease is a corneal lattice dystrophy [7, 12]. Typical diagnostic triad includes progressive bilateral facial paralysis, loose skin (cutis laxa), and lattice corneal dystrophy [1, 12]. The ophthalmologic manifestations include dry eye, irritation, and increased sensitivity to light. Other reported manifestations are exposure keratopathy, dysfunction of the meibomian glands, early cataract development, and an increased risk of secondary chronic open-angle glaucoma [7, 12, 13]. It is believed that increased intraocular pressure (IOP) is due to the effect of mutated gelsolin in the trabecular muscle cells; no amyloid deposits were found in the trabecular meshwork [13]. Facial paralysis and loose skin affect eye lid closure and can cause ectropion leading to corneal exposure with corneal ulcers, sometimes requiring plastic surgery [14, 15].

## 2. Case Report

A 53-year-old female patient was referred to our department by severe dry eye and incomplete eyelid closure. At the age of 45 years the only symptom was a mild dry eye medicated with ocular lubricants, but then she began to realize she had difficulty in frowning. She was diagnosed with Meretoja's syndrome at the age of 50 after a bilateral facial paresis and the diagnose was confirmed by the presence of gelsolin mutation (G654A). The patient was from the north coast of Portugal and had positive family history: her mother had the disease. She had no knowledge of any ancestor from Finland. When the patient was referred to our Ophthalmology Department she had severe bilateral facial palsy, with significant orbicularis and perioral sagging as well as hypoesthesia of extremities. On neurological examination we observed marked facial diparesis, tongue fasciculations, hypoesthesia in the median nerve territory bilaterally, brisk tendon reflexes, and normal coordination tests. Nerve conduction studies revealed slow (median, ulnar, radial, superficial, and sural peroneal nerves) sensitive and motor nerve fibers conduction. Needle electromyography was normal. Left plantar and palmar sympathetic skin responses were absent on electrical stimulation, indicating sympathetic sudomotor fibers involvement. Heart rate response to deep breathing was unremarkable. Quantitative sensory test revealed an increased sensitivity threshold to the vibratory stimuli and hypersensitivity to heat but normal results to cold stimulation.

**Figure 1:**
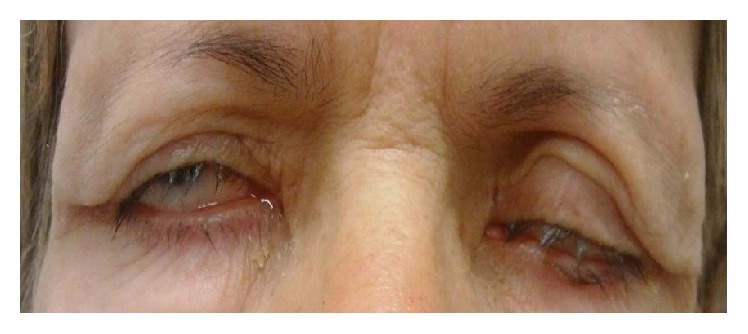
Incomplete eyelid closure.

**Figure 2:**
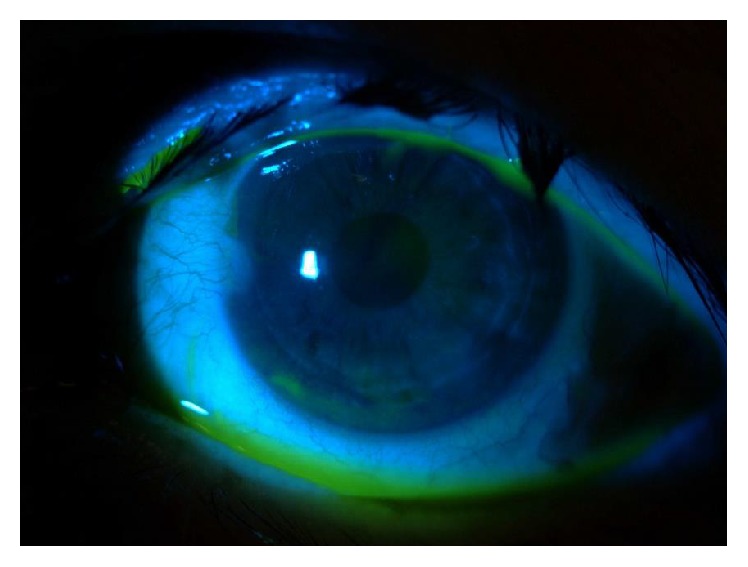
Inferior keratitis.

**Figure 3:**
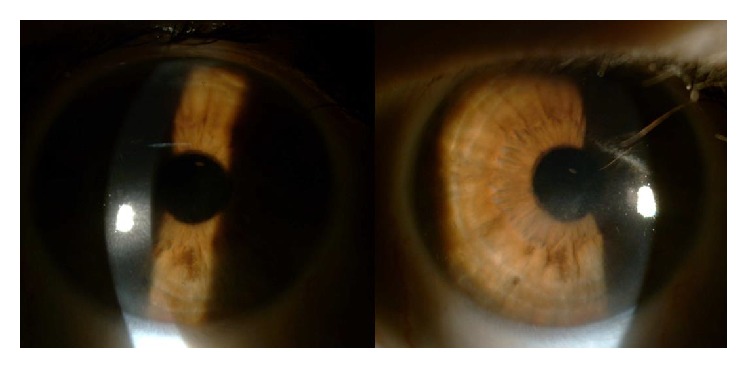
Corneal haze with linear subepithelial opacities in the anterior stroma.

**Figure 4:**
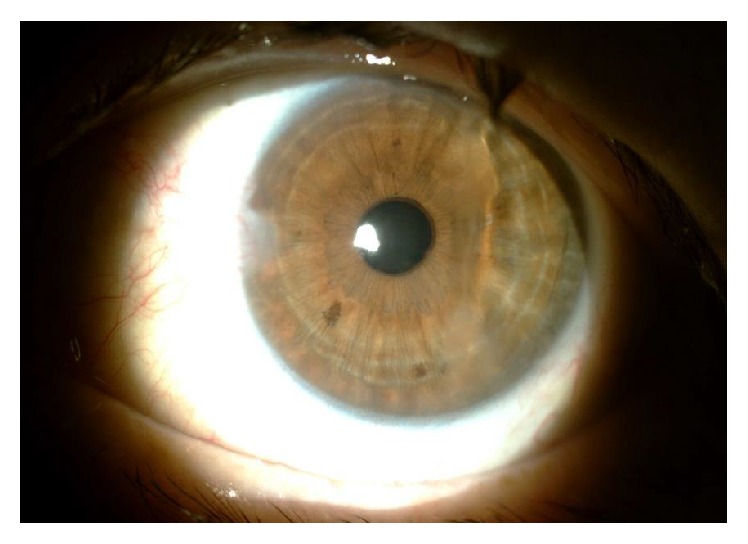
Corneal opacification with neovascularization.

At our observation, she had an incomplete eyelid closure specially in the right eye (Figure 1) and decreased blinking reflex; the best corrected visual acuity (BCVA) was 0.6 bilaterally (decimal scale) and the slit lamp examination showed a diminished tear film break-up time (less than 5 seconds), a diffuse keratitis (Figure 2), corneal haze with linear subepithelial opacities in the anterior stroma of both eyes (Figure 3), and a corneal leukoma with neovascularization in the left eye (Figure 4). Schirmer test was 8 and 9 mm, respectively. IOP was 12 mmHg and 10 mmHg, respectively, and fundus observation showed no alterations. She was medicated with preservative-free lubricants associated with nocturnal complete occlusion of both eyes. Three months later, despite symptomatic improvement, the ocular surface remained quite similar and the patient was treated with topical cyclosporine at the concentration of 0.05%, 1 drop twice daily (twelve hours apart). Ten months after treatment there was an improvement in right eye BCVA (0.8); the slit lamp examination showed bilateral central stromal haze, no keratitis in the right eye, and improvement in the left eye. In the next 3 months there was a worsening of the ocular discomfort and at observation she had a bilateral inferior keratitis with a corneal erosion in the right eye. A temporary lateral tarsorrhaphy was proposed, but the patient refused it. She underwent bilateral lacrimal punctal plugs placement and at the end of the follow-up—6 months after plugs placement—the BCVA was 0.8 in the right eye and 0.6 in the left eye; at slit lamp examination there is no keratitis or corneal erosions and a lower degree of haze and there was some regression of corneal vessels in the left eye. The IOP was 18 mmHg and 16 mmHg, respectively, and fundoscopy was normal.

## 3. Discussion

Meretoja's syndrome is a rare condition with severe and debilitating ocular manifestations and the diagnosis is typically made by observation of the corneal lattice dystrophy [16]. Dry eye syndrome is one of the most common manifestations and is caused by the involvement of several cranial nerves—decreased blink reflex (trigeminal nerve) and weak contraction of the orbicularis muscle (facial nerve). Cutis laxa and peripheral facial paralysis can cause ectropion contributing to a worsening of the problem [7, 12, 13, 15]. Treatment is symptomatic with topical lubricants, anti-inflammatory therapy, hydrophilic contact lenses, and occlusion of the lacrimal ducts with plugs. In the most severe cases surgical treatment is required; eyelid surgery (tarsorrhaphy, ectropion correction) or even penetrating keratoplasty is some of the options, but most often with poor prognosis [13, 15]. Ocular symptoms are the first to appear and our role as ophthalmologists is essential in the diagnosis, treatment, and monitoring of ocular alterations. These patients should have regular ophthalmologic checkups to maintain a healthy ocular surface and avoid severe corneal damage with irreversible visual loss, also allowing early detection of other severe complications such as glaucoma.
